# Effect of a Decellularized Tendon-Based Mitral Annuloplasty Ring on Regurgitation Suppression in Degenerative Mitral Regurgitation Model: An *In Vitro* Pulsatile Circulation Study

**DOI:** 10.1093/icvts/ivag040

**Published:** 2026-02-06

**Authors:** Ikuo Katayama, Shinya Imai, Yusei Okamoto, Kiyotaka Iwasaki

**Affiliations:** Cooperative Major in Advanced Biomedical Sciences, Graduate School of Advanced Science and Engineering, Waseda University, Tokyo 162-8480, Japan; Cooperative Major in Advanced Biomedical Sciences, Graduate School of Advanced Science and Engineering, Waseda University, Tokyo 162-8480, Japan; Department of Integrative Bioscience and Biomedical Engineering, Graduate School of Advanced Science and Engineering, Waseda University, Tokyo 162-8480, Japan; Department of Modern Mechanical Engineering, Graduate School of Creative Science and Engineering, Waseda University, Tokyo 169-8555, Japan; Cooperative Major in Advanced Biomedical Sciences, Graduate School of Advanced Science and Engineering, Waseda University, Tokyo 162-8480, Japan; Department of Integrative Bioscience and Biomedical Engineering, Graduate School of Advanced Science and Engineering, Waseda University, Tokyo 162-8480, Japan; Department of Modern Mechanical Engineering, Graduate School of Creative Science and Engineering, Waseda University, Tokyo 169-8555, Japan; Institute for Medical Regulatory Science, Waseda University, Tokyo 162-8480, Japan; Waseda Research Institute for Science and Engineering, Waseda University, Tokyo 169-8555, Japan

**Keywords:** biological ring, mitral annuloplasty, decellularized biological tissue, *ex vivo* model

## Abstract

**Objectives:**

Conventional annuloplasty rings used in mitral valve repair (MVr) are made of metal or synthetic polymers, which may increase infection risk. This study aimed to develop a mitral annuloplasty ring using decellularized tissue and evaluate its ability to suppress regurgitation in a degenerative mitral regurgitation (DMR) model.

**Methods:**

A 4 mm diameter annuloplasty ring was created using decellularized bovine tendon. Porcine mitral valve complexes (including the annulus, leaflets, chordae tendineae, and papillary muscles) were obtained from a slaughterhouse. The annulus was enlarged by 4 mm, and the 2 chordae tendineae of the posterior leaflet (P2) were severed. The DMR model, integrated into a pulsatile flow simulator, was repaired using a commercial—Physio II, Colvin-Galloway (CG) Future, Tailor band, and a decellularized tendon-based ring. Regurgitation control and effective mitral valve area (MVA) were compared (*n* = 6 for each group).

**Results:**

The regurgitation rate of the DMR model was 52.3 ± 3.4%, consistent with severe MR. Post-MVr with each ring, the regurgitation rates were 14.9 ± 3.1% (Physio II), 14.5 ± 1.1% (CG Future), 16.4 ± 1.7% (Tailor band), and 15.5 ± 3.0% (decellularized tendon-based biological ring). All of these rates were significantly reduced, with no significant differences among them. Effective MVA was comparable across groups: 2.46 ± 0.28 cm^2^ (Physio II), 2.33 ± 0.54 cm^2^ (CG Future), 2.28 ± 0.12 cm^2^ (Tailor band), and 2.27 ± 0.53 cm^2^ (decellularized tendon-based biological ring).

**Conclusions:**

The decellularized tendon-based annuloplasty ring demonstrated functional performance comparable to that of current mitral annuloplasty devices.

## Graphical abstract

**Figure:**
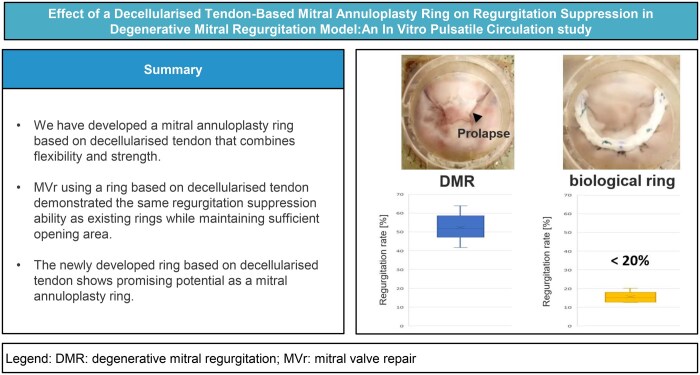

## INTRODUCTION

Mitral regurgitation (MR) is the most common valvular heart disease affecting approximately 24.2 million people worldwide. Its prevalence continues to increase with the ageing global population. Correspondingly, the number of mitral valve surgeries has increased, with mitral valve repair (MVr) accounting for approximately 75% of these procedures.^1–4^ MR is a known risk factor for mitral valve infectious endocarditis. In such cases, MVr has been reported to yield favourable outcomes compared with MVR in selected patients, although this remains debated because of possible selection bias in the literature.^5^

Annular dilation is commonly observed during MVr for degenerative MR (DMR).^6^ In addition to correcting leaflet prolapse, remodelling annuloplasty is essential for preventing recurrent MR by reshaping the mitral valve annulus with the use of an annuloplasty ring.^7^^,^^8^ However, artificial rings are less suitable in certain cases, such as active infective endocarditis, where the risk of reinfection is a concern, or in paediatric patients, where cardiac growth must be accommodated. Therefore, the use of artificial materials is preferably avoided in these situations. Autologous pericardium strips^9^^,^^10^ or rolls^11^^,^^12^ are often used as biological rings and sutured to the mitral annulus. Although the performance of autologous pericardium in MVr and tricuspid annuloplasty has been reported to be comparable to that of existing rings, several challenges remain. These include variability in the quality and quantity of the pericardium that can be harvested due to individual differences, technical difficulty in harvesting during repeat surgeries in children due to adhesions or inflammation-related thickening, and the intraoperative complexity of preparing pericardial rolls. To address these limitations, the development of a decellularized tissue-based mitral annuloplasty ring capable of remodelling the dilated annulus may offer key advantages in reducing infection risk and improving surgical versatility. This study aimed to develop a decellularized tissue-based biological ring and evaluate its feasibility for reducing MR in a DMR model using a pulsatile flow and pressure circulatory simulator.

## METHODS

### Ethical statement

Ethical approval was deemed unnecessary for this study as it was conducted *ex vivo*.

### Preparation of a biological ring

**Figure 1.**
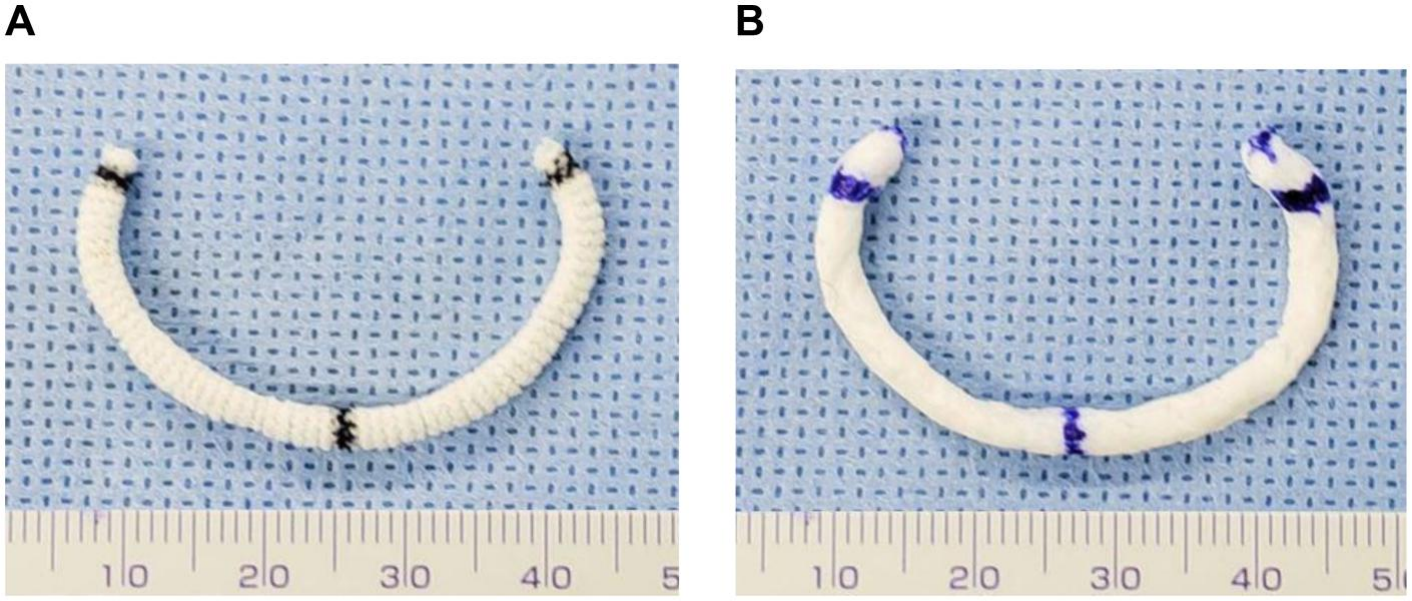
A Decellularized-Tissue-Based Ring of the Same Size as the Existing Ring. (A) Tailor flexible partial band. (B) A decellularized tissue-based flexible ring.

To fabricate the decellularized tissue-based annuloplasty ring, we selected bovine tendon because of its flexibility and resistance to tearing or cutting along the fibre direction. The tendon is longitudinally flexible and sufficiently durable for suturing, even at a diameter comparable to that of a commercially available tailored flexible annuloplasty band (approximately 4 mm). Based on the design of the Tailor Band (**Figure 1A**), we developed a prototype of a flexible, decellularized tendon-based partial ring (**Figure 1B**). The decellularized tendon-based biological ring was designed as a flexible, partial type band, similar to the Tailor band, because it is intended for younger patients with infective endocarditis or paediatric cases, in whom preserving physiological annular motion and minimizing excessive remodelling are essential.

### Preparation of decellularized tissue-based ring

The decellularization process of bovine tendons was based on previous studies^13^^,^^14^ and optimized in our laboratory. Briefly, tendons were decellularized by pulsatile circulation of physiological saline solution with 1 wt% sodium deoxycholate (Sigma) at a mean flow rate of 5 L/min and a mean pressure of 80 mm Hg. Microwave irradiation was applied simultaneously, while maintaining the circulating solution temperature below 37°C. The tissues were then treated with benzonase nuclease (Merck). The combination of pulsatile flow and pressure perfusion of detergent with microwave irradiation was effective in removing cellular components even in relatively thick connective tissues, while preserving the extracellular matrix collagen fibre architecture and mechanical integrity, both of which are critical for maintaining ring flexibility and tensile strength.

To verify the efficacy of decellularization, the residual DNA content was quantified using the PicoGreen DNA assay kit (Invitrogen) showing less than 50 ng of DNA per mg dry weight, which is the generally accepted safety threshold for decellularized biomaterials.^15^ Histological analyses with haematoxylin-eosin (H&E) staining confirmed the absence of cellular nuclei and the preservation of collagen architecture.

### Mechanical strength of decellularized biological rings

A decellularized tendon-based biological ring with a thickness of 4 mm was tested by suturing it with a single stitch of 1 Polysorb (COVIDIEN, Dublin, Ireland). The tissue and sutures were chucked in the grips of a tensile test system (AG-5KNX; SHIMADZU, Tokyo, Japan). The test conditions were as follows: the distance between the tissue clamps was set to 15 mm, the distance between the tissue and threaded clamps was set to 45 mm, and the tissue length was set to 15 mm. A pre-load of 0.01 N was applied to align the collagen of the tissue. The ring was then pulled at a tensile speed of 300 mm/min until rupture to measure its ultimate tensile strength and maximum stiffness.

### Degenerative MR model preparation

**Figure 2.**
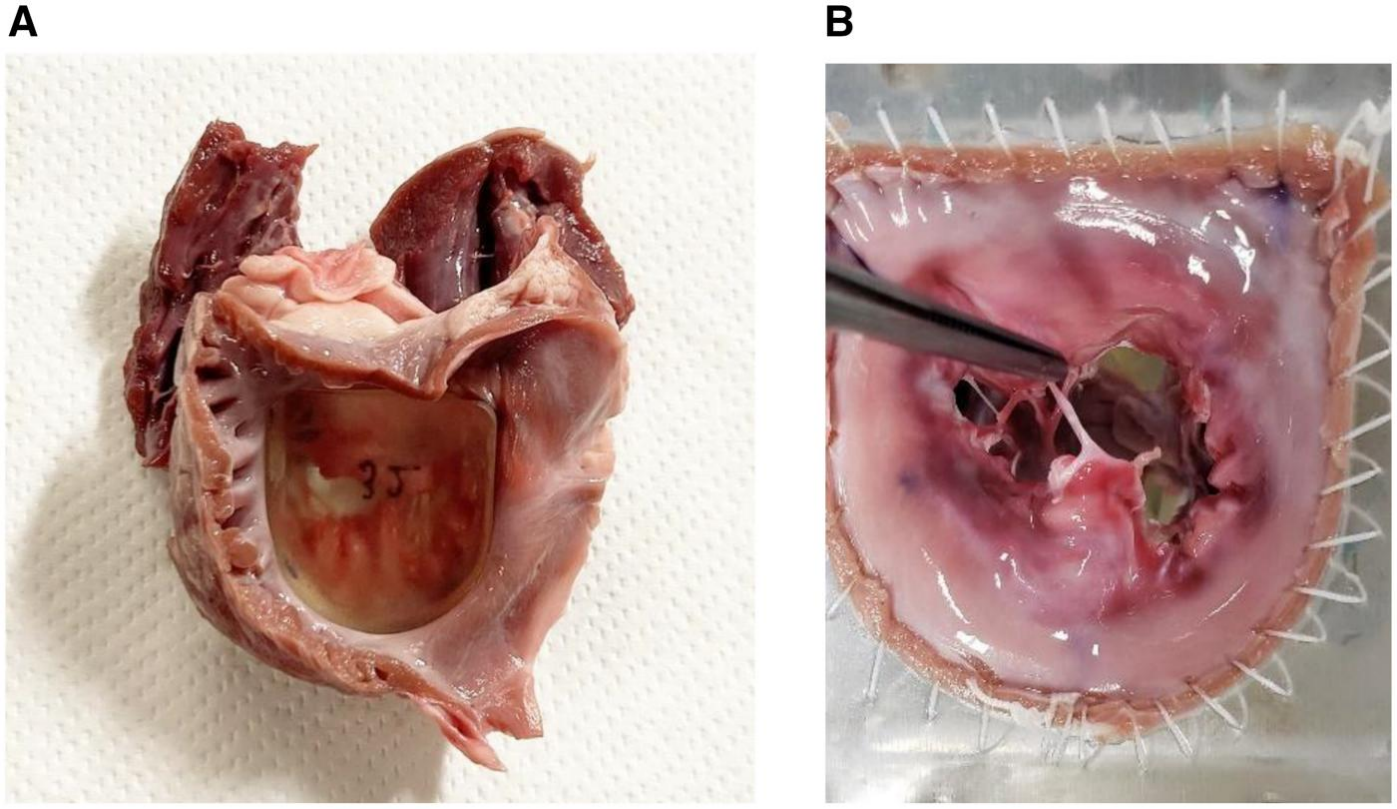
Preparation of a DMR Model Using a Porcine Mitral Valve Complex. (A) Mitral complex with a dilator inserted. (B) Mitral complex sutured to the original silicone sheet.

Porcine hearts were obtained from an abattoir and the mitral valve complex was excised from the left atrium, leaving a 10 mm segment of the mitral annulus intact, including the mitral valve, chordae tendineae, and papillary muscles. To simulate clinical annular dilation, we first injected 0.1% collagenase (C6885-500MG, Sigma-Aldrich, St. Louis, MO, United States) into 5 locations: the bilateral annular commissures and 3 points on the posterior leaflet annulus, inducing minimal degeneration of the annulus tissue. We then inserted an original 3D-printed dilator with a size variation of 2 sizes up (4 mm) from the measured intercommissural distance, incubating it at 37°C for 10 min to create a model of annular dilation (**Figure 2A**). The mitral valve complex with its annulus expanded using a dilator was sutured to an original flexible silicone sheet and incorporated into a pulsatile circulatory simulator. To create the DMR model, one chorda from the anterior papillary muscle and one from the posterior papillary muscle attached to the P2 segment were transected to induce a flail posterior leaflet (**Figure 2B**).

For the repair procedure, the length of the artificial chordae was determined by referencing the adjacent normal chordae to restore physiological coaptation height and leaflet motion. Following chordal reconstruction, annuloplasty was performed using 4 types of rings: Physio II (32 mm, 34 mm), CG Future (32 mm, 34 mm), Tailor band (31 mm, 33 mm), and a decellularized tendon-based biological ring (31 mm, 33 mm). Three commercially available annuloplasty rings with distinct rigidity and geometry were selected for comparison: Physio II (semi-rigid, full ring), CG Future (semi-rigid, partial ring), and Tailor band (flexible, partial band). These devices are widely used in current MVr practice and represent a broad spectrum of stiffness and geometric profiles.

Three samples of each size were used in the study. These sizes corresponded to the actual annular dimensions of the porcine valves employed. Larger or smaller decellularized tendon-based biological rings can be fabricated using the same molding process, and the present study utilized these representative sizes to ensure consistent experimental comparison.

### Comparison of performances of annuloplasty rings using a pulsatile circulation simulator

**Figure 3.**
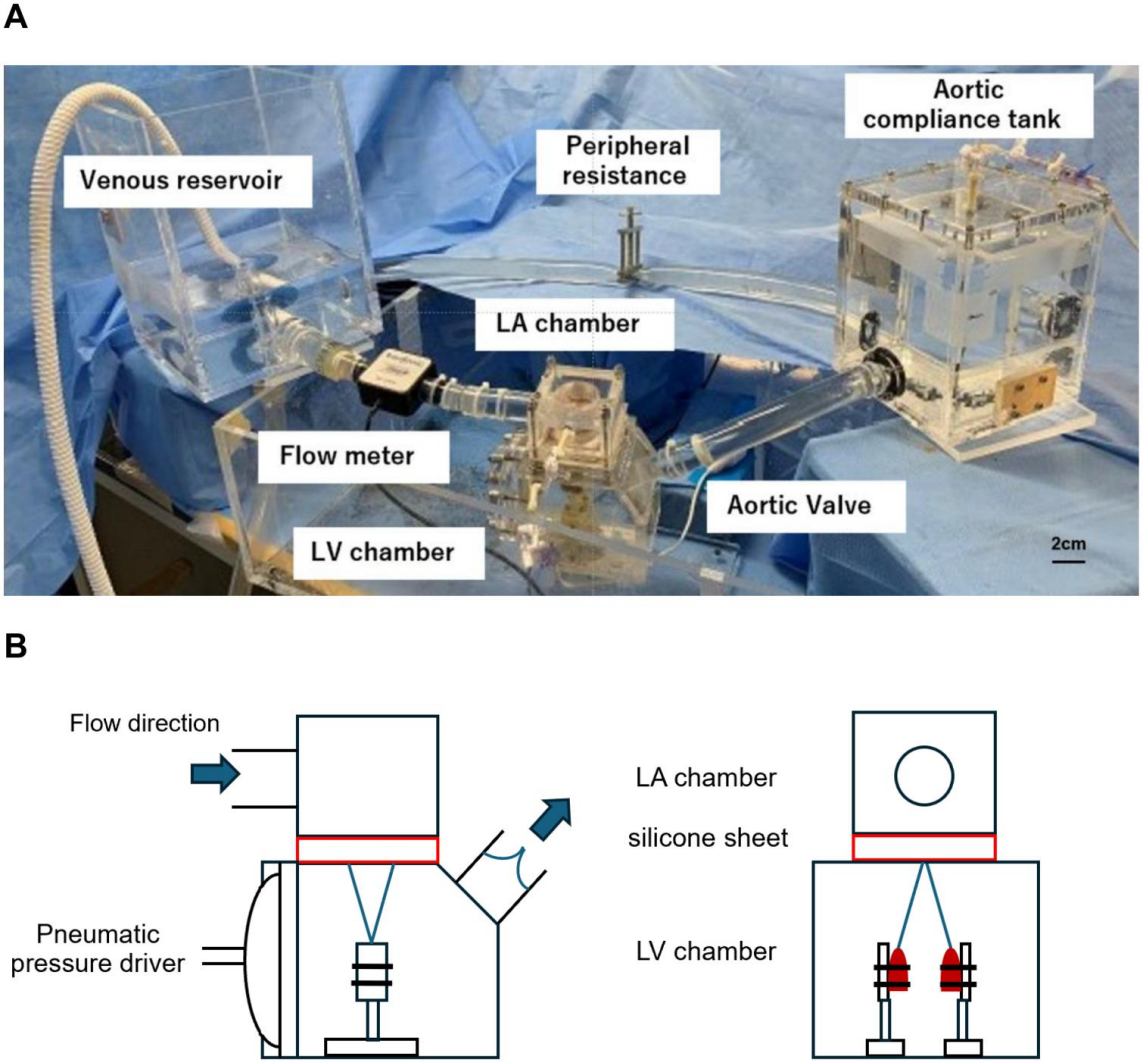
A Pulsatile Flow and Pressure Circulation Simulator. (A) Overall view of the pulsatile circulation simulator incorporating the porcine mitral complex. (B) A cross-sectional images of LA-LV chamber.

**Figure 4.**
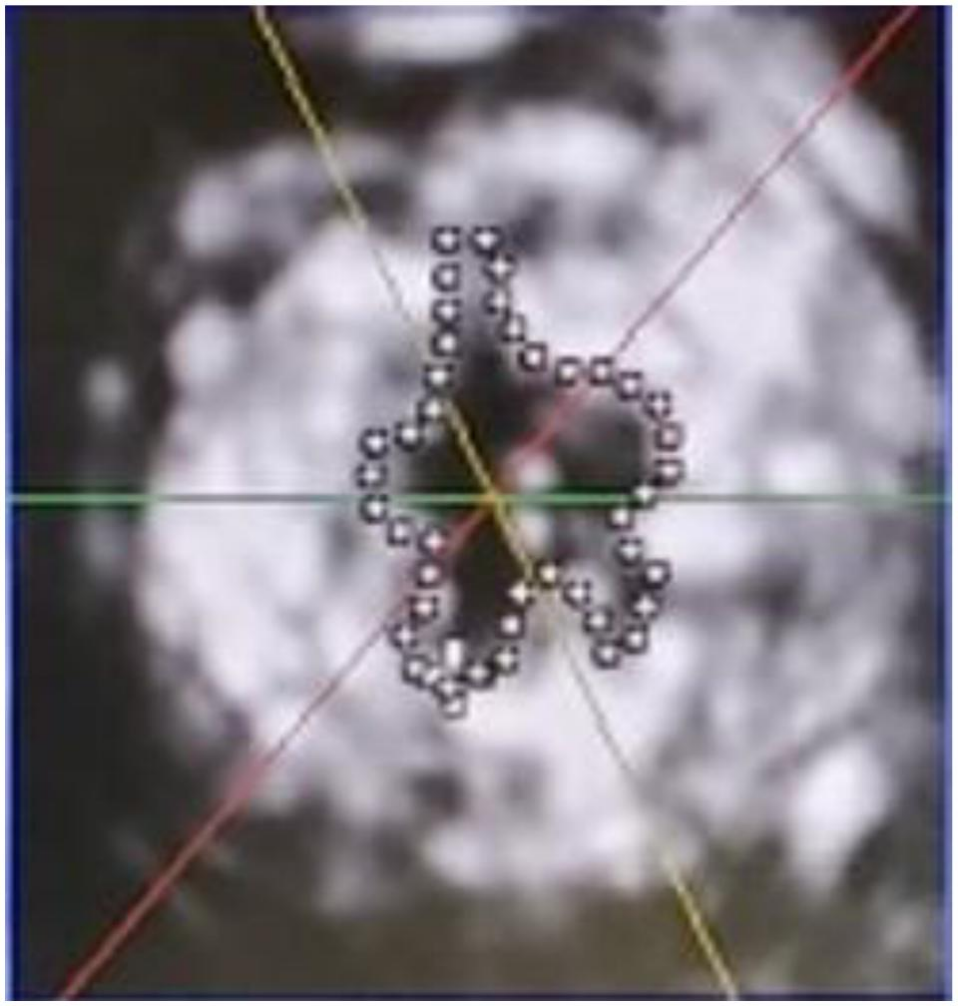
Measurement of Effective Mitral Valve Area (Using the Area Trace Method)

A pulsatile circulation simulator was developed on previous studies.^16^ A porcine mitral valve sutured to a silicone sheet was positioned between the left atrial (LA) and left ventricular (LV) chambers. The flow across the mitral valve was measured using an ultrasonic flow probe (ME-PXN ME19PXN325; Transonic, Ithaca, NY, United States) positioned upstream of the LA chamber (**Figure 3A and B**). LV and aortic pressures were measured using pressure transducers (PXMK10200; TruWave, Edwards Life sciences, Irvine, CA, United States). Haemodynamic data were collected using LabView software (National Instruments, Austin, United States) and subsequently analysed using MATLAB (MathWorks, Natick, United States) to calculate the forward flow (valve inflow) and backflow (valve regurgitation) averaged from 6 continuous waveforms. The measurement was conducted under the following conditions: cardiac output 4.0 L/min, aortic pressure 120/80 mm Hg, heart rate 70 beats/min, and systolic blood fraction 35%. The valve orifice area was measured using an echocardiographic system (Epic CVx3D; Philips, Amsterdam, Netherlands) equipped with a transthoracic probe (X5-1). The maximum mitral valve area (MVA) during diastole was determined from the upper LA chamber using the area-trace method (**Figure 4**).

After preparing the DMR models, regurgitation reduction and mitral valve effective orifice area were compared using Physio II, CG Future, Tailor band, and a decellularized tendon-based biological ring. This was performed in parallel with the reconstruction of a pair of chordae tendineae from the anterior and posterior papillary muscles using artificial chordae tendineae (CV4 polytetrafluoroethylene (PTFE) (Gore-Tex. Flagstaff, AZ, United States)). Six DMR models underwent sequential mitral annuloplasty using 4 different rings (Physio II, CG Future, Tailor band, and the decellularized tendon-based biological ring) after sizing the anterior mitral leaflet with a standard sizer. Three models were tested with 31-32 mm rings and 3 with 33-34 mm rings. The order of ring implantation was not fixed and varied among specimens. Each procedure was performed independently with re-suturing and re-measurement to minimize potential order bias.

### Statistical methods

All values were analysed with SPSS Statistics version 26 (IBM Corp., Tokyo, Japan) and expressed as mean ± SD. A 1-way repeated-measures analysis of variance (ANOVA) was performed with ring type as the within-subject factor. When a significant main effect was detected, pairwise comparisons were conducted using the Bonferroni adjustment. A 2-sided *P*-value of <.05 was considered statistically significant. In addition, given the small sample size (*n* = 6) and the presence of repeated measurements within the same model, the Friedman test was applied as a non-parametric alternative to the repeated-measures ANOVA for the analysis of MVA.

## RESULTS

### Mechanical strength of the decellularized biological ring

The sutures used to secure the ring were initially 2-0 Ti-Cron (0.30-0.339 mm in diameter, tensile strength 26.3 N as specified in the catalogue listed in the catalogue), which are commonly used in clinical settings. However, because these sutures broke prematurely during tensile testing, they were replaced with thicker 1 Polysorb sutures (0.500-0.599 mm in diameter; tensile strength 37.3 N). The early failure of Ti-Cron was attributed to the intentionally high traction load applied to evaluate the mechanical limit of the materials. In contrast to the Tailor band, in which the polyester velour cuff ruptured, the decellularized tendon-based ring remained intact, with only the suture failing under strong traction.

**Figure 5.**
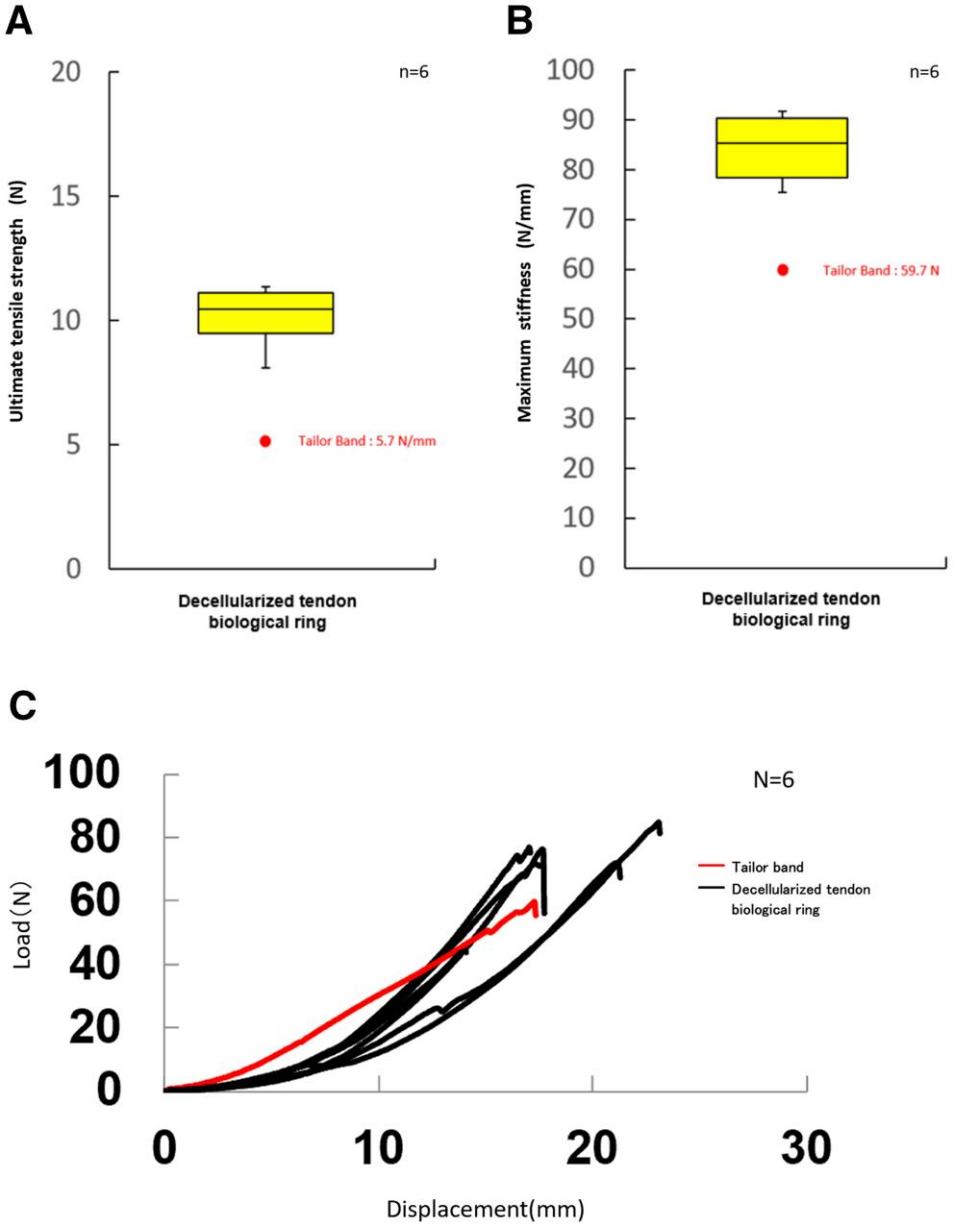
Mechanical Tensile Testing of the Biological Ring and the Tailor Band. Comparison of ultimate tensile strength and maximum stiffness between the polyester velour cuff material of the tailor band and decellularized bovine tendon. (A) Ultimate tensile load. The polyester velour cuff of the tailor band tore before suture rupture, whereas the decellularized tendon-based biological ring remained intact until the suture failed. (B) Maximum stiffness. The biological ring exhibited higher stiffness values across all samples. (C) Load-stroke curve. The biological ring showed a steeper slope, indicating greater stiffness and resistance to deformation compared with the tailor band.

Sutures were tied to both the Tailor band and the decellularized tendon-based biological ring and subjected to tensile loading until failure (**Figure 5A and B**). Because the Tailor band is a commercial product, the mechanical strength of the polyester velour cuff is subjected to strict quality control. Therefore, one representative sample of the Tailor band was tested and compared with the decellularized tendon-based biological ring. In the Tailor band, the polyester velour cuff tore before the suture itself ruptured, whereas in the decellularized tendon-based biological ring, the suture failed while the ring remained intact. This distinct difference in failure mode indicates that the decellularized tendon-based biological ring exhibits greater resistance to suture traction than the Tailor band. Furthermore, the load-stroke curve (**Figure 5C**) demonstrated a steeper slope for the decellularized tendon-based biological ring compared with the Tailor band, indicating higher stiffness. The decellularized tendon-based biological ring maintained its configuration and resisted deformation under traction. No tearing or shrinkage was observed during high-tension suturing, and minimal displacement occurred even at higher loads, reflecting the superior mechanical integrity of the decellularized tendon-based biological ring for annular fixation.

### Regurgitation control ability after MVr using various rings in the DMR model

**Figure 6.**
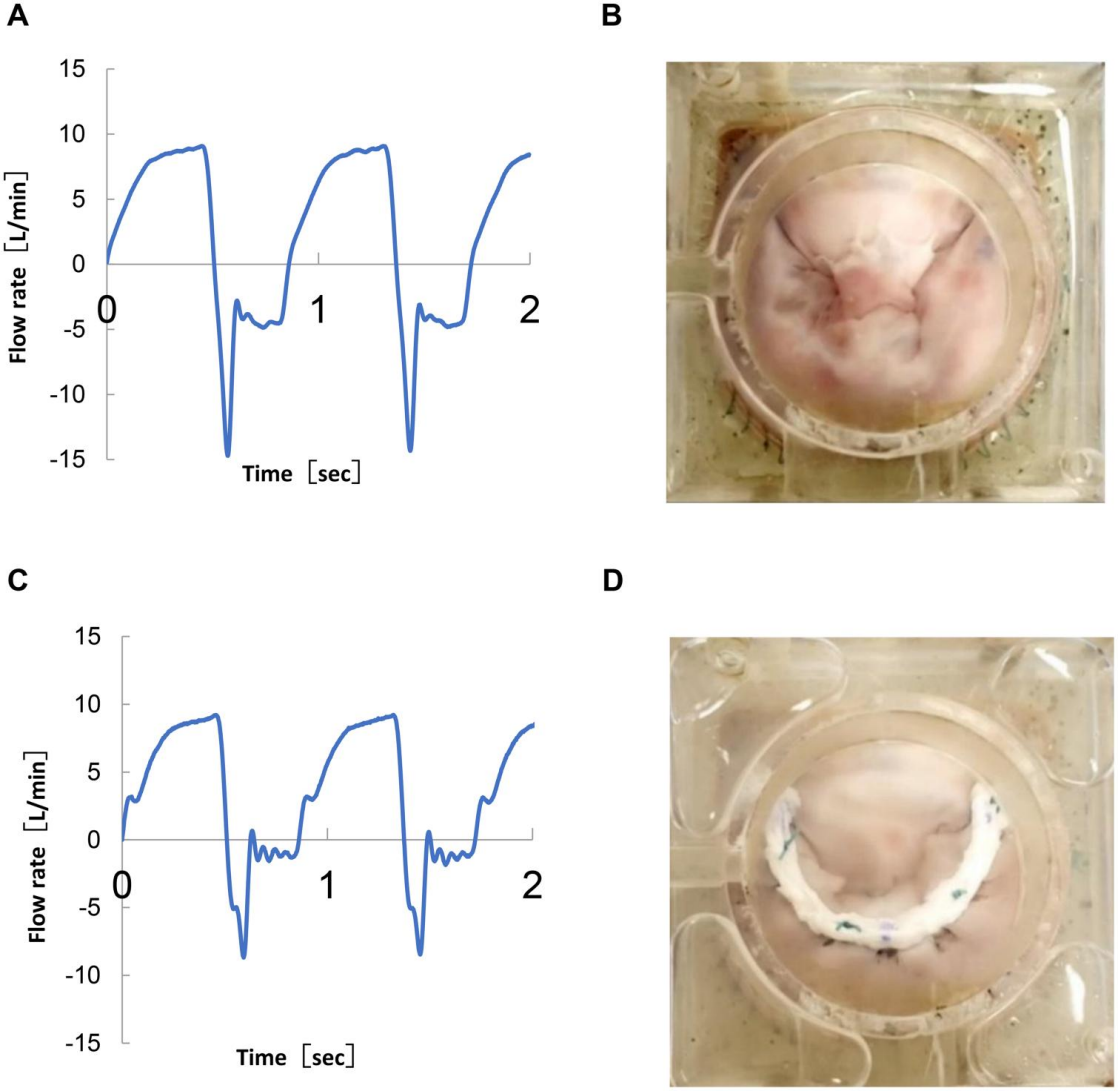
Haemodynamic Data Before and After MVr for the DMR Model. (A) Flow waveform before MVr. (B) Appearance of the DMR model. (C) Flow waveform after MVr using a decellularized tendon-based biological ring. (D) Appearance after MVr using a decellularized tendon-based biological ring.

Mitral valve repair using a decellularized tendon-based biological ring was performed in the DMR model (**Figure 6A and B**). A pair of artificial chordae tendineae was reconstructed from the anterior and posterior papillary muscles, and the model was integrated into a pulsatile circulation simulator to evaluate regurgitation control (**Figure 6C**). The decellularized tendon-based biological ring was sutured to the porcine mitral annulus (**Figure 6D**).

In severe DMR models exhibiting more than 50% regurgitation, the regurgitation rates after MVr were 14.9 ± 3.1% for the Physio II ring, 14.5 ± 1.1% for the CG Future ring, 16.4 ± 1.7% for the Tailor band, and 15.5 ± 3.0% for the decellularized tendon-based biological ring.

**Figure 7.**
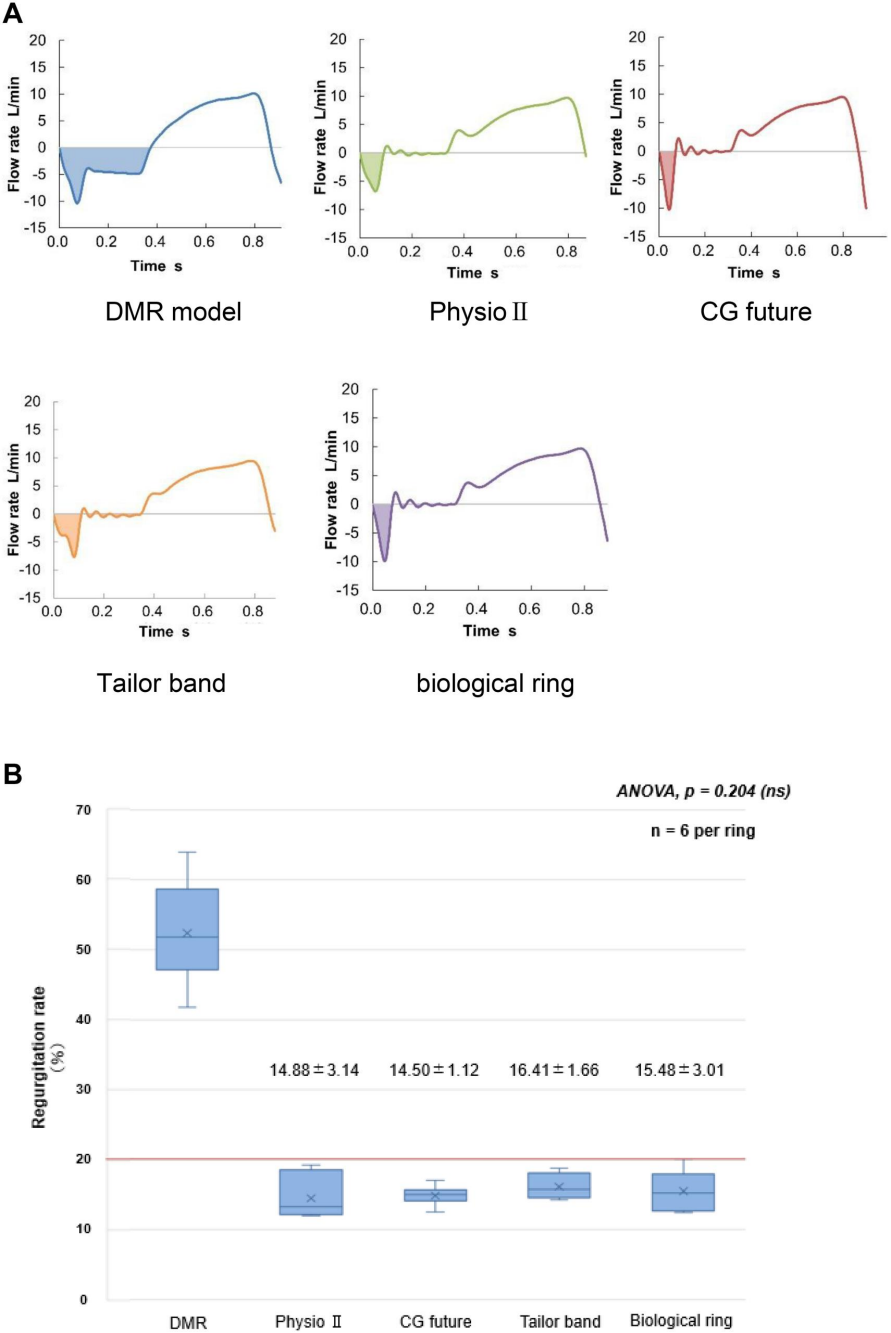
Hemodynamic Waveforms and Regurgitation Rates Following Mitral Valve Repair Using 4 Annuloplasty Devices. (A) Pulsatile flow waveforms recorded in the degenerative mitral regurgitation (DMR) model before repair (left) and after mitral valve repair (MVr) using each annuloplasty ring (Physio II, CG Future, Tailor flexible band, and the decellularized tendon-based biological ring). All waveforms were obtained under identical flow and pressure conditions within the pulsatile circulation simulator, demonstrating effective restoration of physiologic flow patterns following MVr. (B) Quantitative comparison of regurgitation rates following MVr using the 4 ring types in 6 DMR models. Data are shown as mean ± SD (*n* = 6). Statistical analysis was performed using 1-way repeated-measures ANOVA (Friedman test approximation), which showed no significant differences among the 4 rings (*χ*^2^(3) = 4.60, *P* = .204; all pairwise *P* > .7). All rings achieved effective suppression of regurgitation to below 20%, confirming equivalent repair performance. “ANOVA, *P* = .204 (ns)” is indicated in the figure panel.

A 1-way repeated-measures analysis of variance showed no significant differences in regurgitation rates among the 4 ring types (*P *= .204) (**Figure 7**). All rings reduced regurgitation to below 20%.

### Mitral valve area

After performing MVr with each ring type, the MVA was measured using the area-trace method with 3D echocardiography (Epic CVx3D; Philips, Amsterdam, Netherlands). Among the partial rings, the Tailor band showed an MVA of 2.28 ± 0.12 cm^2^, while the decellularized tendon-based biological ring showed an MVA of 2.27 ± 0.53 cm^2^.

Given the small sample size, a Friedman test revealed no significant differences in MVA among the 4 ring types (*χ*^2^(3) = 1.20, *P* = .753). Pairwise comparison using the Wilcoxon signed-rank test showed no significant difference between the Tailor band and the decellularized tendon-based biological ring (*P* = 1.000).

In addition, the MVA of the CG Future was 2.33 ± 0.54 cm^2^ and that of the Physio II was 2.46 ± 0.28 cm^2^, with no significant differences compared with the decellularized tendon-based biological ring (all *P* > .7).

## DISCUSSION

We successfully developed a DMR model using porcine mitral valve complexes with an annular enlargement suitable for ring implantation. The mitral annulus has a saddle-shaped 3D structure, and during MR, the posterior leaflet side between the trigones typically undergoes the most significant dilation.^17^^,^^18^ Collagenase was locally injected into the posterior annulus to create a clinically relevant DMR model. This method selectively weakens the annulus, while preserving the integrity of the surrounding tissues, allowing it to withstand multiple sutures and facilitating both leaflet prolapse and annular enlargement. This approach enables consistent lesion modelling for testing various rings, thereby supporting a comparative performance evaluation. We developed a decellularized tendon-based biological ring that is both durable and flexible enough to be sutured onto the mitral annulus, and tested it in a pulsatile circulation simulator incorporating the DMR model. Our results showed that the decellularized tendon-based biological ring was comparable to existing mitral annuloplasty rings in terms of regurgitation control and haemodynamic performance.

Mitral valve area is generally preferred over valve replacement for the treatment of degenerative MR and selected cases of infective endocarditis, because of its association with superior postoperative survival, preserved left ventricular function, and reduced thromboembolic risk.^5^ However, in cases of active infective endocarditis or small paediatric annuli, the use of artificial materials can be problematic, creating a need for biocompatible, flexible, and infection-resistant alternatives. In such settings, the decellularized tendon-based biological ring offers distinct potential advantages. Its collagen-based scaffold provides sufficient mechanical strength to support annular plication while avoiding rigid fixation, thereby allowing dynamic annular contraction and expansion. This may contribute to more physiological leaflet motion, potentially enhancing long-term valve durability and reducing leaflet stress. Moreover, its biological nature may reduce bacterial adhesion and biofilm formation compared with synthetic materials. A previous study on the implantation of a decellularized tendon for anterior cruciate ligament reconstruction in a rat model demonstrated the *in vivo* repopulation of autologous cells.^19^ Therefore, the decellularized tendon is expected to resist infection, further supporting its use in MVr.

Although both autologous and bovine pericardium have been employed for biological annuloplasty,^9–12^^,^^20–24^ their use may be limited by tissue thinness, as well as inflammation or adhesions in certain patients, particular when autologous pericardium is used. Preparation of pericardial strips or twisted bands requires trimming and shaping, making the outcome surgeon-dependent and less reproducible. In contrast, the decellularized tendon-based biological ring provides a standardized and reproducible alternative, comparable to commercial flexible bands in term of ease of sizing and implantation. Furthermore, the tendon material offers superior form stability and resistance to deformation compared with pericardium. These characteristics suggest that a tendon-derived biological ring may overcome some of the technical and structural limitations associated with pericardial annuloplasty. The biological ring exhibited greater resistance to suture traction than the Tailor band, reflecting its robustness against tearing rather than increased rigidity. The ring retains sufficient flexibility to conform to the physiological saddle shape of the mitral annulus, thereby preserving normal annular dynamics.

In relation to the applicability of our mechanical testing results, we compared the measured tensile strength values with force levels typically encountered during mitral annuloplasty and *in vivo* annular loading. Previous experimental studies have reported that the average suture pull-out force during mitral annuloplasty ranges from approximately 2 to 4 N, and that the radial annular forces observed *in vivo* are generally below 2 N.^25^ In our study, the ultimate tensile load of the decellularized tendon-based biological ring exceeded 40 N, which is an order of magnitude greater than the forces typically observed under surgical and physiological conditions. This substantial safety margin suggests that the biological ring possesses sufficient mechanical robustness for clinical application.

Relative mitral stenosis (MS) caused by mitral annuloplasty can negatively impact the postoperative quality of life (QOL). MVr often involves procedures such as leaflet resection, suturing, artificial chordae reconstruction, and annuloplasty. These surgical interventions could theoretically lead to some degree of MVA reduction, potentially resulting in relative MS, reduced exercise tolerance, and lowered QOL. Mesana *et al*^26^ reported that annular plication using particularly small rings is a risk factor for relative MS. In the present study, a comparison between the Tailor band and the decellularized tendon-based biological ring showed that the decellularized tendon-based biological ring maintained an equivalent or larger mitral valve orifice area. This finding suggests that the decellularized tendon-based biological ring allows sufficient annular plication while remaining flexible and partial, thereby posing a low risk of relative MS.

### Limitations

This study has some limitations. First, it was based on the experimental results of annular plication using a biological ring to control regurgitation caused by posterior leaflet (P2) prolapse. The effectiveness for anterior leaflet lesions remains unknown. However, because annular dilation primarily occurs on the posterior side, regardless of whether the lesion is on the anterior or posterior leaflet, it is reasonable to assume that if the posterior annulus exhibits a plication effect similar to that of the existing partial rings, comparable control of regurgitation could be achieved.

Second, complete blinding of the investigator was not feasible, because the operator was necessarily aware of which annuloplasty ring was being implanted during each experiment. Therefore, the potential for observer bias cannot be entirely excluded. Nevertheless, all comparative experiments were performed under identical conditions using same DMR model and pulsatile circulation simulator, and quantitative assessments were based on objective image and flow analyses. We, therefore, believe that the influence of subjective bias was minimized. This should, however, be acknowledged as one of the limitations of the present study.

Third, this study was conducted entirely in an *ex vivo* setting; thus, *in vivo* animal studies are required to verify biocompatibility, host responses, and long-term durability. Although the *ex vivo* functional evaluation of the decellularized tendon-based biological ring showed comparable performance to existing rings, the effects of host-tissue interaction, remodelling, and potential calcification remain to be investigated in long-term *in vivo* models.

Finally, because the present study employed single-pull tensile testing, cyclic fatigue behaviour, and long-term mechanical durability of the biological ring remain to be determined. Future *in vitro* and *in vivo* studies are warranted to evaluate its mechanical stability under repetitive loading conditions that mimic physiological cardiac cycles, as well as to assess the *in vivo* remodelling of the decellularized bovine tendon-based biological ring.

Despite these limitations, the decellularized tendon-based biological ring developed in this study demonstrated regurgitation control comparable to that of commercial mitral annuloplasty rings. It is flexible and durable enough to withstand multiple sutures and conforms to the physiological motion of the mitral annulus. These characteristics are particularly promising for paediatric patients, where future growth must be considered, and for selected cases of infective endocarditis, where avoidance of artificial materials is desirable.

## CONCLUSIONS

We successfully developed a decellularized tendon-based biological ring that demonstrated regurgitation control comparable to that of existing mitral annuloplasty rings. This biological ring is flexible yet sufficiently strong to maintain its structural integrity during multiple suture placements and conforms to the physiological motion of the mitral annulus.

The decellularized tendon-based biological ring represents a potential advancement in MVr, combining biological compatibility with mechanical reliability.

## DATA AVAILAVILITY

The data underlying this article will be shared on reasonable request to the corresponding author.

## References

[1] Dziadzko V , Clavel M-A, Dziadzko M, et al Outcome and undertreatment of mitral regurgitation: a community cohort study. Lancet. 2018;391:960-969. 29536860 10.1016/S0140-6736(18)30473-2 PMC5907494

[2] Singh JP , Evans JC, Levy D, et al Prevalence and clinical determinants of mitral, tricuspid, and aortic regurgitation (the Framingham Heart Study). Am J Cardiol. 1999;83:897-902. 10190406 10.1016/s0002-9149(98)01064-9

[3] Nkomo VT , Gardin JM, Skelton TN, et al Burden of valvular heart diseases: a population-based study. Lancet. 2006;368:1005-1011. 16980116 10.1016/S0140-6736(06)69208-8

[4] Enriquez-Sarno M , Akins CW, Vahanian A. Mitral regurgitation. Lancet. 2009;373:1382-1394. 19356795 10.1016/S0140-6736(09)60692-9

[5] Feringa HH , Shaw LJ, Poldermans D, et al Mitral valve repair and replacement in endocarditis: a systematic review of literature. Ann Thorac Surg. 2007;83:564-570. 17257988 10.1016/j.athoracsur.2006.09.023

[6] Kapadia S , Krishnaswamy A, Wierup P, et al Mitral annulus three-dimensional configuration and size in normal, degenerative, and functional mitral regurgitation. J Cardiovasc Comput Tomogr. 2020;14:S88.

[7] Kasegawa H , Kamata S, Ida T, et al Physiologic remodeling annuloplasty to retain the shape of anterior leaflet: new concept in mitral valve repair. J Heart Valve Dis. 1997;6:604-607. 9427128

[8] Carpentier A. Cardiac valve surgery—the “French correction”. J Thorac Cardiovasc Surg. 1983;86:323-337. 6887954

[9] De La Zerda DJ , Cohen O, Marelli D, et al Long-term results of mitral valve repair using autologous pericardium annuloplasty. J Heart Valve Dis. 2008;17:10-15. 18365563

[10] Delmo Walter EM , Siniawski H, Ovroutski S, et al Mitral valve growth after posterior annular stabilization with untreated autologous pericardial strip in children with mitral valve insufficiency. Ann Thorac Surg. 2010;90:1577-1585; discussion 1585. 20971267 10.1016/j.athoracsur.2010.06.084

[11] Matsuyama K , Matsumoto M, Sugita T, et al Long-term results of mitral valve reconstruction using autologous pericardium for annular support in active infective endocarditis. Eur J CardioThorac Surg. 2005;27:884-888.

[12] Miura T , Obase K, Matsumaru I, et al Very long-term outcomes of twisted auto-pericardial mitral annuloplasty. Gen Thorac Cardiovasc Surg. 2020;68:1113-1118. 32124200 10.1007/s11748-020-01324-3

[13] Iwasaki K. Innovative bioreactor technologies produced a completely decellularized and preendothelialized functional aortic valve. In: Proc*eedings* of 12th Int Conf on Biomed Eng. CD-ROM, Institute of Physics Publishing, 2005.

[14] Itoh M , Itou J, Okazaki K, et al Estimation failure risk by 0.5-mm differences in autologous hamstring graft diameter in anterior cruciate ligament reconstruction: a meta-analysis. Am J Sports Med. 2024;52:535-543. 36876736 10.1177/03635465221150654

[15] Crapo PM , Gilbert TW, Badylak SF. An overview of tissue and whole organ decellularization processes. Biomaterials. 2011;32:3233-3243. 21296410 10.1016/j.biomaterials.2011.01.057 PMC3084613

[16] Arita M , Tono S, Kasegawa H, et al Multiple purpose simulator using natural porcine valve. Asian Cardiovasc Thorac Ann. 2004;12:350-356. 15585707 10.1177/021849230401200415

[17] Salati M , Scrofani R, Santoli C. Posterior pericardial annuloplasty: a physicological correction. Eur J Cardiothorac Surg. 1991;5:226-229; discussion 229. 1859660 10.1016/1010-7940(91)90168-j

[18] Scrofani R , Moriggia S, Salati M, et al Mitral valve remodeling: long-term results with posterior pericardial annuloplasty. Ann Thorac Surg. 1996;61:895-899. 8619713 10.1016/0003-4975(95)01139-0

[19] Itoh M , Imasu H, Takano K, et al Time-series biological responses toward decellularized bovine tendon graft and autograft for 52 consecutive weeks after rat anterior cruciate ligament reconstruction. Sci Rep. 2022;12:6751. 35468916 10.1038/s41598-022-10713-y PMC9038763

[20] Borghetti V , Campana M, Scotti C, et al Biological versus prosthetic ring in mitral valve repair: enhancement of mitral annulus dynamics and left-ventricular function with pericardial annuloplasty at long term. Eur J Cardiothorac Surg. 2000;17:431-439. 10773567 10.1016/s1010-7940(00)00344-4

[21] Bevilacqua S , Cerillo AG, Gianetti J, et al Mitral valve repair for degenerative disease: is pericardial posterior annuloplasty a durable option. Eur J Cardiothorac Surg. 2003;23:552-559. 12694775 10.1016/s1010-7940(02)00867-9

[22] Victor S , Nayak VM, Truly Flexible D-shaped autogenous pericardial ring for mitral annuloplasty. Ann Thorac Surg. 1993;56:179-180. 8328859 10.1016/0003-4975(93)90435-k

[23] Pomerantzeff PM , de Almeida Brandão CM, Albuquerque JM, et al Mitral valve annuloplasty with a bovine pericardial strip—18-year results. Clinics (Sao Paulo). 2005;60:305-310. 16138237 10.1590/s1807-59322005000400008

[24] Kuralay E. Mitral ring annuloplasty by biological material. Turk Gogus Kalp Damar Cerrahisi Derg. 2022;30:645-648. 36605320 10.5606/tgkdc.dergisi.2022.23578 PMC9801468

[25] Siefert AW , Pierce EL, Lee M, et al Suture forces in undersized mitral annuloplasty: novel device and measurements. Ann Thorac Surg. 2014;98:305-309. 24996707 10.1016/j.athoracsur.2014.02.036 PMC4109808

[26] Mesana TG , Lam BK, Chan V, et al Clinical evaluation of functional mitral stenosis after mitral valve repair for degenerative disease: potential affect on surgical strategy. J Thorac Cardiovasc Surg. 2013;146:1418-1425. 24075470 10.1016/j.jtcvs.2013.08.011

